# An *NPPB* Promoter Polymorphism Associated With Elevated N‐Terminal pro–B‐Type Natriuretic Peptide and Lower Blood Pressure, Hypertension, and Mortality

**DOI:** 10.1161/JAHA.116.005257

**Published:** 2017-03-24

**Authors:** Sara B. Seidelmann, Orly Vardeny, Brian Claggett, Bing Yu, Amil M. Shah, Christie M. Ballantyne, Elizabeth Selvin, Calum A. MacRae, Eric Boerwinkle, Scott D. Solomon

**Affiliations:** ^1^ Cardiovascular Division Brigham and Women's Hospital Boston MA; ^2^ Division of Cardiovascular Imaging Department of Radiology Brigham and Women's Hospital Boston MA; ^3^ Departments of Pharmacy and Medicine University of Wisconsin‐Madison Madison WI; ^4^ Epidemiology, Human Genetics & Environmental Sciences UTHealth School of Public Health Houston TX; ^5^ Methodist DeBakey Heart Center Baylor College of Medicine Houston TX; ^6^ The Johns Hopkins Institute for Clinical and Translational Research Baltimore MD; ^7^ Human Genome Sequencing Center Baylor College of Medicine Houston TX

**Keywords:** blood pressure, hypertension, mortality, natriuretic peptide, NPPB, N‐terminal pro–B‐type, polymorphism, rs198389, single nucleotide polymorphism, Genetic, Association Studies, Blood Pressure, Hypertension, Mortality/Survival, Risk Factors

## Abstract

**Background:**

Elevated B‐type natriuretic peptide (BNP) levels are associated with heart failure and increased mortality in the general population. We investigated rs198389, a functional variant in the promoter region of the BNP gene (*NPPB*), in patients from the Atherosclerosis Risk in Communities Study to investigate associations with N‐terminal pro‐BNP (NT‐proBNP) levels and outcomes.

**Methods and Results:**

A total of 11 361 black and white patients with rs198389 genotyping attended visit 1 (aged 45–64 years; 1987–1989), with follow‐up visits occurring every 3 years (visit 2–visit 4, 1990–1999), followed by visit 5 (2011–2013). NT‐proBNP levels were measured at visits 2, 4, and 5. At visit 2, the GG genotype (frequency 18%) was associated with a 41% higher mean plasma level of NT‐proBNP compared with the AA genotype (frequency 34%), with intermediate values observed in AGs (*P*=4.2×10^−52^). The GG genotype was associated with reduced systolic blood pressure (−1.6 mm Hg, *P*=0.006), diastolic blood pressure (−1 mm Hg, *P*=0.003), antihypertension medication use (odds ratio, 0.85; 95% CI, 0.74–0.97 [*P*=0.02]), and hypertension (odds ratio, 0.81; 95% CI, 0.72–0.92 [*P*=0.002]) compared with the AA genotype with intermediate values in AGs. These relationships persisted throughout subsequent visits. After a median follow‐up of 23 years, there were 4031 deaths. With and without covariate adjustment, the GG genotype was associated with modestly lower mortality (hazard ratio, 0.86; 95% CI, 0.78–0.95), primarily reflective of cardiovascular death (hazard ratio, 0.75; 95% CI, 0.61–0.92), and increased residual lifespan of 8 months from 50 years of age (*P*=0.02) versus AAs.

**Conclusions:**

The rs198389 G allele in the *NPPB* promoter is associated with elevated levels of NT‐proBNP throughout adult life, reduced blood pressure, hypertension and cardiovascular mortality, and increased lifespan.

## Introduction

B‐type natriuretic peptide (BNP), a member of the natriuretic peptide (NP) family, plays a critical role in the regulation of circulatory volume status, plasma renin‐aldosterone concentrations, natriuresis, and ultimately the maintenance of blood pressure (BP) levels.^1^ Elevated circulating levels of BNP stimulate natriuresis and diuresis as well as promote peripheral vasodilation. In this way, BNP is thought to counterbalance the negative effects of intravascular volume expansion by encouraging excretion of fluid volume and attenuating the elevation of mean arterial pressure. Plasma BNP and N‐terminal proBNP (NT‐proBNP) have become established biomarkers to facilitate the diagnosis and prognosis of heart failure (HF), and have been associated with cardiovascular morbidity and mortality in the general population.^2^

Several common single nucleotide polymorphisms (SNPs) have previously been described in the *NPPB* gene that encodes for the BNP protein. Specifically, rs198389 has been established as a functional variant in the gene's promoter region, disrupting a transcriptionally active enhancer box. Meirhaeghe et al^3^ showed that the G allele was associated with a 1.8‐fold higher level of promoter activity versus the A allele in cultured cells.^3^ At least 7 independent racially diverse human population studies have shown that the minor allele of rs198389 SNP is associated with elevated mean levels of NT‐proBNP and/or BNP^3^, ^4^, ^5^, ^6^, ^7^, ^8^, ^9^ with similar effect size observed for NT‐proBNP and BNP levels.^6^ The minor allele of rs198389 (G allele; higher BNP levels) has been associated with reduced ventricular dysfunction following coronary artery bypass grafting,^10^ reduced hospital readmissions, and lower E/E^1^ in patients after myocardial infarction (MI),^8^ and a lower risk of developing type 2 diabetes mellitus.^3^, ^11^, ^12^ The minor allele of rs198389 was recently found to be modestly associated with reduced systolic BP (SBP) in a meta‐analysis of African Americans.^7^ Minor alleles of SNPs in the *NPPA* gene, associated with both increased atrial NP and BNP levels, unlike specific variants in the *NPPB* gene, have previously been associated with lower BP and incident hypertension^8^, ^13^ in primarily white individuals. Previous genetic association studies looking at cardiovascular outcomes have been small in size or with limited duration of follow‐up.

In the present study, we independently investigated a single SNP, the rs198389 functional variant in the *NPPB* promoter region, in order to characterize associated phenotypes in 11 361 black and white patients who attended visit 1 of the Atherosclerosis Risk in Communities (ARIC) study. We hypothesized that the rs198389 variant, which has been associated with increased *NPPB* transcription, may be associated with NT‐proBNP levels across age and different stimuli as well as with hypertension and mortality. Genotypic associations with outcomes were examined, adjusting for known predictors and confounders including age, race, and body mass index (BMI).

## Methods

### Study Design and Study Population

The ARIC study is an ongoing, prospective observational trial of atherosclerosis risk factors in 4 US communities (Forsyth County, NC; Jackson, MS; suburbs of Minneapolis, MN; and Washington County, MD) originally comprised of 15 792 men and women, aged 45–64 years recruited between 1987 and 1989 (visit 1).^14^ Participants were initially examined every 3 years, with a second examination in 1990–1992, a third in 1993–1995, a fourth in 1996–1998, and a fifth in 2011–2013. The institutional review board at each participating site approved the study protocol, and informed consent was obtained in writing at each examination.

### Identification of Cardiovascular Outcomes and Death

Outcomes evaluated in this study were death subsequent to the first visit until the end of 2013 (median 23‐year follow‐up) and cardiovascular or non–cardiovascular death until the end of 2011. The methods utilized for quality control, detection, and adjudication of events have been previously presented.^15^ Trained abstractors accrued information about all out‐of‐hospital deaths via death certificates, hospitalized patients, physician questionnaires, and next‐of‐kin interviews. We defined cardiovascular death from *International Classification of Diseases, Ninth Revision* (*ICD‐9*) codes 390–459, or *ICD‐10* codes I00–I99. Non–cardiovascular death was defined as any death excluding cardiovascular disease death and unknown death (death *ICD‐9* codes 799, or *ICD‐10* codes R99) or death without *ICD* codes recorded from the fourth visit (1996–1998) until the end of 2011. Previous MI was defined as self‐reported history of MI at visit 1; previous HF was defined as self‐reported history of HF at visit 1.^16^, ^17^

### Genotyping

Genotyping within the ARIC study was performed on the Affymetrix 6.0 DNA microarray (Affymetrix, Santa Clara, CA) and analyzed with the Birdseed variant‐calling algorithm. Quality control criteria were used to filter SNPs containing possible errors. SNPs were excluded from analysis based on previously established thresholds for SNP call rates, Hardy–Weinberg *P* values, and minor allele frequencies.^18^ rs198389 was imputed using IMPUTE2 with the full 1000 Genomes data set (phase 1, version 3) as the reference. Whole genome sequencing data were available for a random sample of 1860 black and 1705 white ARIC study participants. Imputation data were referenced against whole genome sequencing for concordance. While the imputation quality was high in whites (95% concordance compared with whole genome sequencing) it was low in blacks (45% concordance). Therefore, genotypic information was derived directly from whole genome sequencing data for the black study participants.

### Measurement of NT‐proBNP Levels

Blood samples from ARIC visits 2, 4, and 5 were obtained and plasma stored centrally at −80°C. NT‐proBNP was quantified using an electrochemiluminescent immunoassay (Roche Diagnostics) with a lower detection limit of 5 pg/mL.^19^ Patients with NT‐proBNP levels below the lower limit of detection were assigned values equal to half of the lower limit of detection.^19^ The variability in NT‐proBNP concentrations related to frozen storage and freeze‐thaw cycles^20^ as well as interassay coefficient and reliability coefficient of variation have been previously described.^19^ Genotype‐adjusted NT‐proBNP was created by subtracting the predicted log‐NTproBNP levels for each genotype at rs198389 from basal NT‐proBNP levels at visit 2.

### Statistical Analysis

Participants were excluded from the analysis if they restricted use of their DNA (n = 44). Baseline characteristics are summarized using descriptive statistics for continuous variables and number counts and percentages for categorical variables. Linear regression and chi‐square tests for trend were used to test for a relationship between continuous or categorical baseline characteristics and rs198389 genotype. Restricted cubic spline regression models were used to flexibly model the relationship between log‐transformed NT‐proBNP and continuous exposure variables age, SBP, estimated glomerular filtration rate (eGFR), and BMI. These relationships were estimated separately for each genotype group via interaction terms, and a likelihood ratio test was used to assess equality of the genotype‐specific relationships. Beta coefficients (per 1‐SD) were calculated from regression models including age, SBP, BMI, eGFR, and genotype. The analyses of the association of rs198389 with outcomes was stratified or adjusted for self‐reported race and for the first 10 principal components from EIGENSTRAT.^21^ Linear regression for trend was used to test for a relationship between continuous or categorical BP characteristics and rs198389 genotype adjusting for age, sex, race, and the first 10 principal components. SBP was adjusted for +15 mm Hg and diastolic BP +10 mm Hg if the patient was taking antihypertension medication (assessed by self‐reported questionnaire at each visit). Incident risk was calculated for death and cardiovascular and noncardiovascular death. Follow‐up time was calculated as the time from the first examination (or second examination if NT‐proBNP levels were included in the Cox model) to the time of the first event. Hazard ratios (HRs) were calculated using Cox proportional hazards regression. Three multivariate models investigating the association between rs198389 genotype and outcomes were created: first, a base model stratified for age, race, and sex and adjusted for the first 10 principal components. Model 1 includes the base model with further adjustment for diabetes mellitus, BMI, total cholesterol, high‐density lipoprotein cholesterol, tobacco use, and prior HF at visit 1; model 2 begins follow‐up at visit 2, adjusting for genotype‐adjusted NT‐proBNP levels at visit 2 and all variables from model 1 as collected at visit 2. *P* values are calculated using both additive models (*P* for trend) and recessive models for comparison. Kaplan–Meier failure curves were calculated for each outcome. We used Kaplan–Meier curves with participant age on the *x* axis in order to estimate “residual lifespan” from age 50, up to a maximum of 42 years (ie, age 92), using the area under the Kaplan–Meier curve.

All data were analyzed using STATA version 14.0 (College Station, TX). *P*<0.05 was considered statistically significant. No adjustments were made for multiple comparisons.

## Results

**Figure 1:**
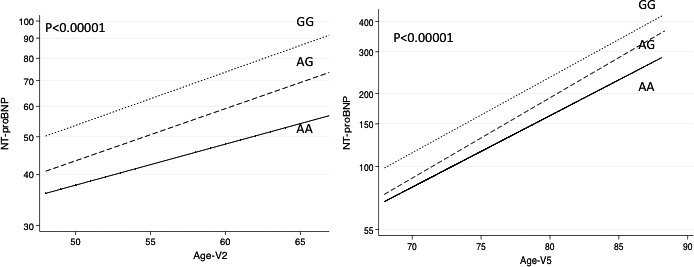
Geometric mean and N‐terminal B‐type natriuretic peptide (NT‐proBNP) vs age by genotype at rs198389 at visit 2 (V2) and visit 5 (V5). *P* value for test of equality between rs198389 genotypes. Adjusted for age, sex, and race.

**Figure 2:**
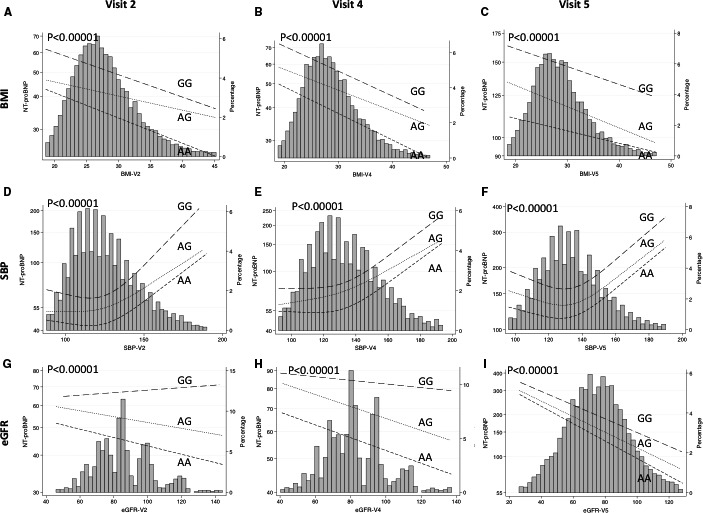
Geometric mean N‐terminal pro–B‐type natriuretic peptide (NT‐proBNP) vs body mass index (BMI; A‐C), systolic blood pressure (SBP; D‐F), and estimated glomerular filtration rate (eGFR; G‐I) by genotype at rs198389 at visit 2 (average age 57), visit 4 (average age 63), and visit 5 (average age 76). The histogram represents the distribution of the variable on the *x* axis and the lines represent spline smoothed mean values of NT‐proBNP within rs198389 genotypes at each *x* axis variable level. *P* value for test of equality between rs198389 genotypes. Adjusted for age, sex, and race; SBP adjusted for +15 mm Hg if taking antihypertension medications.

**Figure 3:**
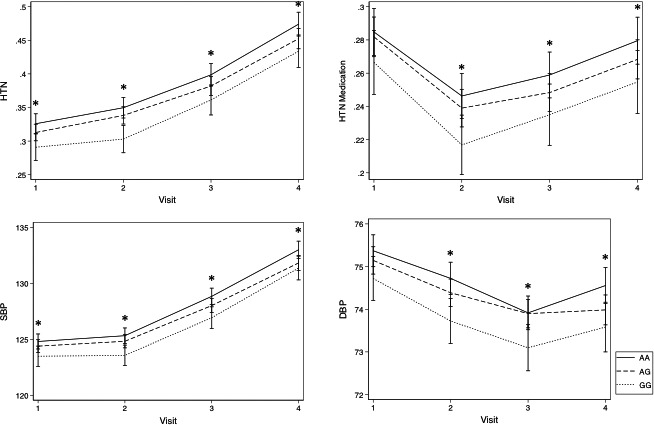
Prevalence of hypertension (HTN; defined as +anti‐HTN medications or blood pressure >140/80 mm Hg), use of anti‐HTN medications, and systolic blood pressure (SBP) and diastolic blood pressure (DBP) adjusted for the use of anti‐HTN medication, at visit 1 (average age 54 years), visit 2 (average age 57 years), visit 3 (average age 60 years), and visit 4 (average age 63 years) in blacks and whites. Means and *P* values adjusted for age, sex, and race. *P≤0.02.

Genotypic frequencies of rs198389 were similar between white and black patients (Table 1). Patients with each genotype were similar in age, sex, BMI, smoking, prevalence of diabetes, HF, and MI (Table 1). Levels of NT‐proBNP were significantly higher in those with the GG and AG genotypes on 3 separate visits that were ≈5 and 14 years apart (visit 2, *P*=4.2×10^−52^; visit 4, *P*=1.5×10^−29^; visit 5, *P*=1.1×10^−16^ [Table 2]) in blacks and whites. As genotypic frequencies were similar and the association with NT‐proBNP levels were consistent in whites and blacks, they were combined for all further analyses, with adjustment for self‐reported race and principal components. For any value of age, BMI, SBP, or eGFR, patients with the AG genotype had intermediate NT‐proBNP levels and those with the GG genotype had the highest NT‐proBNP compared with patients with the AA genotype (*P*<0.00001 for all [Figures 1 and 2]). These relationships were significant at all 3 visits. In order of importance, age, SBP, rs198389 genotype, and BMI, followed by eGFR were most predictive of NT‐proBNP levels based on beta coefficients calculated for 1 SD of each variable at visit 2. eGFR increased in importance in influencing NT‐proBNP levels by visit 5, in order of importance: age, eGFR, rs198389 genotype, SBP, and BMI. The GG genotype was associated with reduced SBP (visit 1–visit 4, *P*=0.038–0.002), diastolic BP (visit 2–visit 4, *P*=0.036–0.003), antihypertension medication use (visit 2–visit 4: odds ratio, 0.85; 95% CI, 0.74–0.98 [*P*<0.03]), and hypertension (visit 1–visit 4: odds ratio, 0.81–0.86; 95% CI, 0.72–0.97, [*P*=0.01–0.002]) compared with the AA genotype with intermediate values observed in AGs (Figure 3; Table S1).

**Table 1:** Characteristics of Patients in the ARIC Study Cohort Stratified by rs198389 Polymorphism in the B‐Type Natriuretic Peptide Gene From Visit 1 (average age 54; n=11 361), visit 2 (average age 57; n=10 931), visit 4 (average age 63; n=8943), and visit 5 (average age 76; n=5017)

Characteristic	Whites				Blacks			
	AA	AG	GG	*P* Trend^a^	AA	AG	GG	*P* Trend^a^
Visit 1								
No. (%)	3201 (34)	4544 (48)	1717 (18)		673 (35)	909 (48)	317 (17)	
Age	54.3±6	54.4±6	54.3±6	0.9	52.9±6	52.8±6	52.8±6	0.9
Men, No. (%)	1461 (46)	2178 (48)	807 (47)	0.2	248 (37)	319 (35)	116 (37)	0.8
Smoking, No. (%)	811 (25)	1140 (25)	389 (23)	0.07	196 (29)	259 (29)	76 (24)	0.1
Diabetes mellitus, No. (%)	285 (9)	371 (8)	160 (9)	0.9	109 (17)	150 (17)	53 (17)	0.8
BMI	26.9±5	27.0±5	27.2±5	0.2	30.0±6	29.6±6	29.8±6	0.4
Total cholesterol	214.3±40	215.0±40	215.7±43	0.2	209.6±40	213.6±42	216.1±44	0.01
HDL‐C	50.7±17	50.6±17	50.1±16	0.8	54.8±17	55.8±18	55.6±18	0.3
Previous HF, No. (%)	111 (4)	182 (4)	64 (4)	0.5	38 (6)	48 (5)	17 (6)	0.9
Previous MI, No. (%)	120 (4)	199 (4)	71 (4)	0.6	19 (3)	42 (5)	9 (3)	0.8
Visit 2								
Diabetes mellitus, No. (%)	359 (11.7)	492 (11.3)	201 (12.1)	0.9	154 (24.5)	199 (23.6)	73 (24.1)	0.7
BMI	27.2±4.8	27.3±5.0	27.5±5.0	0.2	30.5±6.3	29.9±6.1	30.0±6.3	0.07
Total cholesterol	209.3±38.5	209.5±38.5	210.7±40.0	0.2	207.3±40.0	211.0±40.1	207.2±39.0	0.9
HDL‐C	48.7±16.5	48.6±16.5	47.7±16.1	0.3	52.5±15.8	53.8±18.1	52.1±16.3	0.9
Previous HF, No. (%)	117 (3.8)	182 (4.2)	63 (3.8)	0.8	40 (6.2)	50 (5.8)	18 (5.8)	0.8
Previous MI, No. (%)	136 (4.4)	221 (5.1)	79 (4.8)	0.6	20 (3.1)	42 (4.9)	8 (2.6)	0.9
Visit 4								
Diabetes mellitus, No. (%)	352 (13.6)	486 (13.4)	207 (14.8)	0.5	114 (26.1)	142 (24.1)	57 (25.6)	0.7
BMI	28.3±5.3	28.3±5.3	28.3±5.2	0.9	31.2±6.5	30.9±6.6	30.0±6.5	0.03
Total cholesterol	200.9±34.8	201.2±37.7	201.0±36.7	0.6	196.2±37.9	200.0±36.4	199.9±37.1	0.2
HDL‐C	49.0±16.1	49.2±16.4	48.3±16.0	0.7	51.7±16.3	54.1±16.8	54.2±16.7	0.03
Previous HF, No. (%)	176 (5.5)	252 (5.5)	102 (5.9)	0.9	53 (7.9)	88 (9.7)	29 (9.1)	0.8
Previous MI, No. (%)	200 (6.2)	310 (6.8)	111 (6.5)	0.8	39 (5.8)	71 (7.8)	22 (6.9)	0.4
Visit 5								
Diabetes mellitus, No. (%)	405 (29.6%)	598 (30.9%)	245 (31.0%)	0.6	120 (45.8%)	150 (43.6%)	61 (47.7%)	0.8
BMI	28.3±5.4	28.1±5.2	28.2±5.1	0.5	31.2±7.3	30.3±7.0	30.0±7.4	0.07
Total cholesterol	181.5±42.2	180.0±42.2	178.6±40.9	0.3	182.6±39.1	185.9±42.2	180.8±38.5	0.8
HDL‐C	52.1±13.7	51.6±14.4	51.3±13.7	0.5	51.6±11.6	55.7±15.2	53.7±13.6	0.02
Previous HF, No. (%)	109 (7.7)	162 (8.1)	81 (10.0)	0.1	30 (10.6)	53 (14.4)	20 (14.5)	0.2
Previous MI, No. (%)	114 (8.1)	182 (9.1)	67 (8.3)	1.0	22 (7.8)	20 (5.4)	11 (8.0)	0.7

ARIC indicates Atherosclerosis Risk in Communities; BMI, body mass index; HDL‐C, high‐density lipoprotein cholesterol; HF, heart failure; MI, myocardial infarction.

a Adjusted for age and sex.

**Table 2:** Geometric Mean NT‐proBNP Levels in Whites and Blacks From Visit 2 (average age 57; n=10 931), visit 4 (average age 63; n=8943), and visit 5 (average age 76; n=5017)

Characteristic	Whites				Blacks				Combined *P* Value
	AA	AG	GG	*P* Trend^a^	AA	AG	GG	*P* Trend^a^	
Visit 2, No.	3086	4374	1663		640	860	308		
Visit 2, age, y	57±5.7	57±5.7	57±5.6	1.0	56±5.7	56±5.7	56±5.6	0.9	
Visit 2, NT‐proBNP	46 (44–48)	57 (55–59)	64 (61–68)	4.2×10^−37^	29 (27–32)	41 (38–45.0)	55 (48–63)	3.1×10^−15^	6.0×10^−52^
Visit 4, No.	2599	3640	1401		461	613	229		
Visit 4, NT‐proBNP	63 (60–66)	77 (74–80)	86 (81–91)	1.5×10^−22^	32 (28–37)	44 (39–49)	56 (47–66)	1.8×10^−7^	5.2×10^−29^
Visit 5, No.	1412	2005	812		283	367	138		
Visit 5, NT‐proBNP	144 (136–152)	161 (153–169)	204 (189–220)	1.1×10^−13^	83 (71–97)	108 (95–122)	126 (102–155)	1.0×10^−3^	5.4×10^−16^

NT proBNP indicates N‐terminal pro–B‐type natriuretic peptide.

a Adjusted for age and sex.

**Figure 4:**
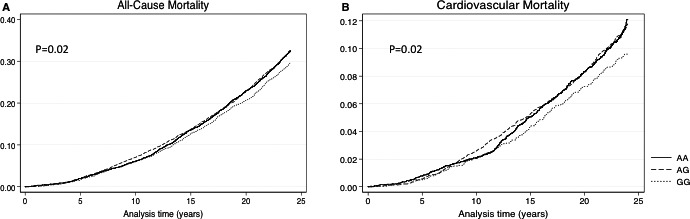
Kaplan–Meier failure curves for all‐cause death (A) and cardiovascular mortality (B) stratified by rs198389 genotype in whites and blacks (N=11 361).

After a median of 23 years of follow‐up, 4031 participants had died. Inheritance of the GG genotype conferred modestly decreased risk of all‐cause mortality in the cohort compared with the AA and AG genotypes (HR, 0.91; 95% CI, 0.83–1.00 [*P*=0.026]; Figure 4A) as well as after multivariate analysis adjusting for known confounders (HR, 0.90; 95% CI, 0.82–1.00 [*P*=0.015]). Cardiovascular death was significantly reduced in patients with the GG genotype in unadjusted and multivariate adjusted models (HR, 0.84; 95% CI, 0.70–1.00 [*P*=0.031] and HR 0.77; 95% CI, 0.63–0.94 [*P*=0.009], respectively) in subanalysis of the total cohort (Figure 4B; Table S2). These relationships remained significant after further adjustment for genotype‐adjusted NT‐proBNP levels (Table S2). After assessment of the 4 individual ARIC centers, the magnitude of effect of the GG genotype was consistently protective from cardiovascular death (Figure S1). Noncardiovascular death was not significantly different in GGs versus AAs. The GG genotype was associated with an increase in residual lifespan of 8 months from 50 years of age in patients compared with AAs (*P*=0.020).

## Discussion

In this large, biracial, prospective cohort study, we show that the rs198389 *NPPB* promoter variant is highly associated with large differences in NT‐proBNP levels in both blacks and whites. The GG genotype conferred 40% higher NT‐proBNP levels compared with AA genotype. The rs198389 variant remained important in predicting NT‐proBNP levels throughout lifetime and stimuli. For any measure of age, BMI, SBP, and eGFR, patients with the AG and GG genotype had progressively higher NT‐proBNP levels compared with those with AA genotype. Patients with the GG genotype had reduced SBP and diastolic BP and were ≈15% less likely to take antihypertension medication and 19% less likely to have the diagnosis of hypertension. After covariate adjustment, there was evidence that the GG genotype was associated with lower mortality, particularly cardiovascular mortality, and was also associated with a significant increase in residual lifespan compared with the AA genotype. These data suggest that genetically determined elevation in natriuretic peptide levels may be associated with benefit.

In this work, we confirm that rs198389 is strongly associated with NT‐proBNP levels in the general population. The relationship of levels with genotype is robust, persisting throughout adult lifespan and varying BMI and renal function. At visit 2, genotype was more important than eGFR and BMI in predicting NT‐proBNP levels but slightly less important than age and SBP. Later in life, at visit 5, age remained most important, followed by eGFR, followed by genotype, SBP, and BMI. It is likely that as hypertension and renal dysfunction become more prevalent in old age, SBP plays a lesser role in influencing NT‐proBNP levels and eGFR becomes a better predictor. Regardless, this work shows that the rs198389 genotype remains significantly associated with levels throughout the ages of ≈45 to 90 years.

We show that the G allele, which results in higher circulating NT‐proBNP levels, is associated with a lower risk of hypertension in whites and blacks. Supportive evidence of the effect of elevated NPs in lowering BP has been shown in animal models. Transgenic mice overexpressing BNP or atrial natriuretic peptide (ANP) are hypotensive in comparison to littermates.^22^, ^23^ Previous human‐based studies also support this finding. In a large meta‐analysis of common variants in the *NPPA‐NPPB* locus, Newton‐Cheh et al^13^ showed that *NPPA* polymorphisms (rs5068 and rs198358) were associated with increased BNP and NT‐proBNP as well as ANP levels and a lower risk of hypertension. In the same study, a variant in *NPPB* (rs632793) was tested and shown to be associated with ANP and BNP levels alone. While rs198389 was not specifically assessed in that study, it has been shown to be in linkage disequilibrium with rs632793 (Spearman correlation coefficient=0.90 in the ARIC cohort).^13^, ^24^ Since *NPPB* and *NPPA* lie in close proximity to each other on the chromosome, and genetic variants in both the *NPPA* and *NPPB* gene (ie, rs5068, rs198358, and rs632793) have been shown to be associated with both BNP and ANP levels, this further supports coordinated transcriptional regulation across the locus. Therefore, it is possible that the antihypertensive response observed in this study is solely attributable to the actions of elevated BNP itself or, perhaps, through altered levels of multiple natriuretic peptides.

We detected a modest, but significant protective effect of the GG genotype from all‐cause mortality in blacks and whites. In a subanalysis of cardiovascular and noncardiovascular death, the rs198389 GG genotype seemed to be primarily protective from cardiovascular death. The G allele of rs198389 has been associated with a reduction in rehospitalization in patients following MI after 1.8 years of follow‐up.^8^ A difference in mortality was not detected, but the study follow‐up was of limited duration. However, taken with our current data, this prior work supports a potential cardioprotective effect for the G allele of rs198389.

**Figure 5:**
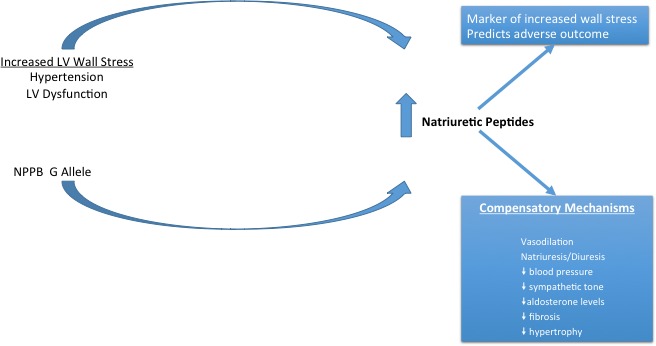
Natriuretic peptide levels are influenced both by genetic factors and conditions that increase cardiac wall stress. Elevation in natriuretic peptides serves as a compensatory mechanism, resulting in vasodilation, natriuresis, and diuresis, with resultant decrease in blood pressure, sympathetic tone, aldosterone, fibrosis, and ventricular hypertrophy. Nevertheless, elevation in natriuretic peptides are markers of increased wall stress and predict adverse outcomes. LV indicated left ventricular; NPPB, B‐type natriuretic peptide gene G allele at rs198389.

Although elevated BNP levels are strongly associated with adverse cardiovascular outcomes, a growing body of evidence from animal studies and population‐based studies (as discussed above), as well as clinical trials, is demonstrating that elevated basal levels of BNP may play a beneficial role (Figure 5). The release of BNP in response to hypertension and elevated SBP is a normal physiological response to restore fluid balance and hemodynamic homeostasis. In this sense, genetically elevated BNP could potentially be beneficial as the system is able to respond distinctively to given stimuli. In contrast, cardiac dysfunction, secondary to an insult such as MI, results in increased preload and afterload, decreased cardiac output and hemodynamic decompensation, and activation of the sympathetic nervous system and renin‐angiotensin‐aldosterone system. Under these circumstances, BNP may increase many‐fold as a compensatory means to restore homeostasis. In this sense, elevation in BNP is a marker of hemodynamic stress and predictive of adverse outcomes. Herein lies the dichotomy of interpreting NP levels and highlights the importance of fine phenotyping genetic variants such as rs198389 that cause large differences in plasma NP levels so that we can better understand this dichotomy.

Evidence from clinical trials has also been generally supportive of a cardioprotective role of increased circulating levels of NPs. Prior clinical studies have shown that infusions of BNP or ANP in patients with HF with reduced ejection fraction are associated with favorable hemodynamic, neurohormonal, and renal effects, although they have not proven effective in reducing death or hospitalization in patients with acute decompensated HF.^25^, ^26^, ^27^, ^28^ In contrast, sacubitril/valsartan, an angiotensin receptor neprilysin inhibitor, that inhibits the breakdown of natriuretic peptides and other vasoactive proteins, was recently shown to lower the risk of death and hospitalization in patients with chronic HF.^29^ Our data support that elevated and sustained levels of NPs throughout adult life may be cardioprotective.

### Study Limitations

Our study has some limitations that are worth noting. First, we do not have measures of other natriuretic peptides including ANP and CNP in order to fully understand the effects of the rs198389 variant on broader natriuretic peptide pathways. Second, we did not assess the relationship of rs198389 in other population studies; however, we did replicate our findings in 2 diverse racial groups, in multiple independent visits, and/or independent study centers. Finally, the association of rs198389 with mortality should be considered as hypothesis generating and consistent with the hypotensive effect of the minor allele; therefore, it should be tested in other populations with large study samples and long‐term outcomes.

## Conclusions

The rs198389 G allele is associated with elevated basal levels of NT‐proBNP across one's lifetime, reduced systolic and diastolic BP and hypertension, protection from cardiovascular mortality and, ultimately, increased lifespan in the ARIC cohort.

## Sources of Funding

The ARIC study is carried out as a collaborative study supported by National Heart, Lung, and Blood Institute contracts (HHSN268201100005C, HHSN268201100006C, HHSN268201100007C, HHSN268201100008C, HHSN268201100009C, HHSN268201100010C, HHSN268201100011C, and HHSN268201100012C). Dr Seidelmann is supported by National Institutes of Health grant number 2T32HL094301‐06.

## Disclosures

None.

## Supporting information


**Table S1.** Systolic and Diastolic Blood Pressure*, Use of Antihypertension (anti‐HTN) Medications and Prevalence of HTN (Defined as +Anti‐HTN Meds or BP >140/80) From Visit 1 (Average Age 54), Visit 2 (Average Age 57), Visit 3 (Average Age 60), Visit 4 (Average Age 63), and Visit 5 (Average Age 76) in Blacks and Whites
**Table S2.** Risk of All‐Cause Mortality, Cardiovascular, and Noncardiovascular Death Based on *ICD* Codes in Patients in the Atherosclerosis Risk in Communities Study Cohort Stratified by rs198389 Polymorphism in the B‐Type Natriuretic Peptide Gene Post Visit 1 (Model 2 is Post Visit 2)
**Figure S1.** Risk of cardiovascular death in patients in the Atherosclerosis Risk in Communities Study cohort with the GG genotype of the rs198389 polymorphism in the B‐type Natriuretic Peptide gene vs GA or AA genotypes by study center.
