# Supplementary material for: An NPPB Promoter Polymorphism Associated With Elevated N‐Terminal pro–B‐Type Natriuretic Peptide and Lower Blood Pressure, Hypertension, and Mortality
Source: J Am Heart Assoc. 2017 Mar 24;6(4):e005257. doi: 10.1161/JAHA.116.005257 (PMC5533018; doi:10.1161/JAHA.116.005257)
Supplement: Supplementary file 1 — Table S1. Systolic and Diastolic Blood Pressure*, Use of Antihypertension (anti‐HTN) Medications and Prevalence of HTN (Defined as +Anti‐HTN Meds or BP >140/80) From Visit 1 (Average Age 54), Visit 2 (Average Age 57), Visit 3 (Average Age 60), Visit 4 (Average Age 63), and Visit 5 (Average Age 76) in Blacks and Whites Table S2. Risk of All‐Cause Mortality, Cardiovascular, and Noncardiovascular Death Based on ICD Codes in Patients in the Atherosclerosis Risk in Communities Study Cohort Stratified by rs198389 Polymorphism in the B‐Type Natriuretic Peptide Gene Post Visit 1 (Model 2 is Post Visit 2) Figure S1. Risk of cardiovascular death in patients in the Atherosclerosis Risk in Communities Study cohort with the GG genotype of the rs198389 polymorphism in the B‐type Natriuretic Peptide gene vs GA or AA genotypes by study center. [file JAH3-6-e005257-s001.pdf]

SUPPLEMENTAL MATERIAL

Table S1. Systolic and Diastolic Blood Pressure*, use of anti-hypertension (anti-HTN) meds and prevalence of HTN (defined as +anti-HTN meds or BP>140/80) from Visit 1 (average age 54), Visit 2 (average age 57), Visit 3 (average age 60), Visit 4 (average age 63) and Visit 5 (average age 76) in blacks and whites.

Characteristic	AA	AG	GG	P-trend
V1 SBP	125.9 ± 0.33	125.6 ± 0.28	124.9 ± 0.45	0.038
V1 DBP	76.1 ± 0.19	75.9 ± 0.16	75.6 ± 0.25	0.074
V1 anti-HTN meds, N (%)	1256 (32.6%)	1697 (31.3%)	589 (29.1%)	
V1 anti-HTN meds, OR (95% CI)	Reference	0.98 (0.89-1.08)	0.92 (0.81-1.04)	0.19
V1 HTN, N (%)	1103 (28.5%)	1536 (28.2%)	542 (26.6%)	
V1 HTN, OR (95% CI)	Reference	0.93 (0.85-1.03)	0.86 (0.76-0.97)	0.012
V2 SBP	126.3 ± 0.34	125.8 ± 0.29	124.7 ± 0.46	0.006
V2 DBP	75.3 ± 0.19	74.9 ± 0.16	74.3 ± 0.26	0.003
V2 anti-HTN meds, N (%)	954 (24.6%)	1303 (23.9%)	441 (21.7%)	
V2 anti-HTN meds, OR (95% CI)	Reference	0.96 (0.87-1.06)	0.85 (0.74-0.97)	0.021
V2 HTN, N (%)	1299 (35.0%)	1766 (33.8%)	595 (30.3%)	
V2 HTN, OR (95% CI)	Reference	0.95 (0.86-1.04)	0.81 (0.72-0.92)	0.002
V3 SBP	129.7± 0.36	128.9± 0.30	128.1 ± 0.49	0.002
V3 DBP	74.5 ± 0.20	74.5 ± 0.17	73.7 ± 0.27	0.036
V3 anti-HTN meds, N (%)	1003 (25.9%)	1354 (24.8%)	478 (23.5%)	
V3 anti-HTN meds, OR (95% CI)	Reference	0.93 (0.85-1.03)	0.85 (0.75-0.98)	0.019
V3 HTN, N (%)	1321 (39.9%)	1790 (38.2%)	644 (36.1%)	
V3 HTN, OR (95% CI)	Reference	0.92 (0.84-1.01)	0.86 (0.76-0.97)	0.010
V4 SBP	133.9 ± 0.38	132.7 ± 0.33	132.5 ± 0.52	0.008
V4 DBP	75.2 ± 0.21	74.6 ± 0.18	74.2 ± 0.28	0.005
V4 anti-HTN meds, N (%)	1083 (28.0%)	1463 (26.8%)	518 (25.5%)	
V4 anti-HTN meds, OR (95% CI)	Reference	0.95 (0.86-1.05)	0.85 (0.75-0.97)	0.020
V4 HTN, N (%)	1444 (47.4%)	1914 (45.2%)	704 (43.4%)	
V4 HTN, OR (95% CI)	Reference	0.90 (0.82-0.99)	0.85 (0.75-0.96)	0.005
V5 SBP	135.8 ± 0.47	133.1± 0.40	134.2 ± 0.62	0.3
V5 DBP	69.3 ± 0.27	68.7 ± 0.23	68.5 ± 0.36	0.059
V5 anti-HTN meds, N (%)	1125 (67.6%)	1519 (65.1%)	626 (67.1%)	

V5 anti-HTN meds, OR (95% CI)	Reference	0.89 (0.78-1.02)	0.99 (0.83-1.18)	0.68
V5 HTN, N (%)	1264 (75.6%)	1694 (72.5%)	692 (73.9%)	
V5 HTN, OR (95% CI)	Reference	0.85 (0.73-0.99)	0.92 (0.77-1.11)	0.2

*Adjusted for age, sex, and race; SBP adjusted for +15mmHg and DBP +10mmHg if patient taking anti-HTN medication. P-values include adjustment for Principle Components.

Table S2. Risk of All cause mortality, Cardiovascular (CV) and non-CV Death based on ICD codes in Subjects in the Atherosclerosis Risk in Communities Study cohort stratified by rs198389 polymorphism in the B-type Natriuretic Peptide gene Post V1 (Model 2 is post V2).

Rs198389	AA	AG	GG	P-value for trend	P-value for recessive model
Total Cohort, N=11,361	3874	5453	2034		
Death, N (%)	1395 (36%)	1958(36%)	678 (33%)		
Death, HR (95% CI), Crude	Reference	1.00 (0.93-1.07)	0.91 (0.83-1.00)	0.069	0.026
Death, HR (95% CI), Age, sex, race	Reference	0.97 (0.90-1.03)	0.90 (0.82-0.99)	0.030	0.049
Death, HR (95% CI), Model 1	Reference	0.97 (0.90-1.04)	0.90 (0.82-1.00)	0.014	0.015
Death, HR (95% CI), Model 2	Reference	0.95 (0.88-1.03)	0.86 (0.78-0.95)	0.005	0.011
CV Death, N (%)	377 (9.7%)	533 (9.8%)	167 (8.2%)		
CV Death, HR (95% CI), Crude	Reference	1.02 (0.88-1.15)	0.84 (0.70-1.00)	0.102	0.031
CV Death, HR (95% CI), Age, sex, race	Reference	0.96 (0.84-1.10)	0.83 (0.69-0.99)	0.059	0.051
CV Death, HR (95% CI), Model 1	Reference	0.97 (0.84-1.11)	0.77 (0.63-0.94)	0.019	0.009
CV Death, HR (95% CI), Model 2	Reference	0.98 (0.84-1.14)	0.75 (0.61-0.92)	0.017	0.005
Non-CV Death, N (%)	797 (20.6%)	1036 (20.4%)	362 (18.8%)		
Non-CV Death, HR (95% CI), Crude	Reference	0.99 (0.90-1.08)	0.90 (0.79-1.01)	0.11	0.066
Non-CV Death, HR (95% CI), Age, sex, race	Reference	0.97 (0.88-1.06)	0.90 (0.79-1.01)	0.088	0.11
Non-CV Death, HR (95% CI), Model 1	Reference	0.97 (0.88-1.06)	0.89 (0.78-1.01)	0.082	0.095
Non-CV Death, HR (95% CI), Model 2	Reference	0.95 (0.85-1.06)	0.84 (0.72-0.97)	0.022	0.032

Model 1= DM, BMI, Tchol, HDL, tobacco use and prior HF; Stratified by age, sex, and race; All models include adjustment for Principle Components

Model 2= Model 1 + genotype-adjusted logNT-proBNP

Figure S1. Risk of Cardiovascular Death in Subjects in the Atherosclerosis Risk in Communities Study cohort with the GG genotype of the rs198389 polymorphism in the B-type Natriuretic Peptide gene versus GA or AA genotypes by study Center.

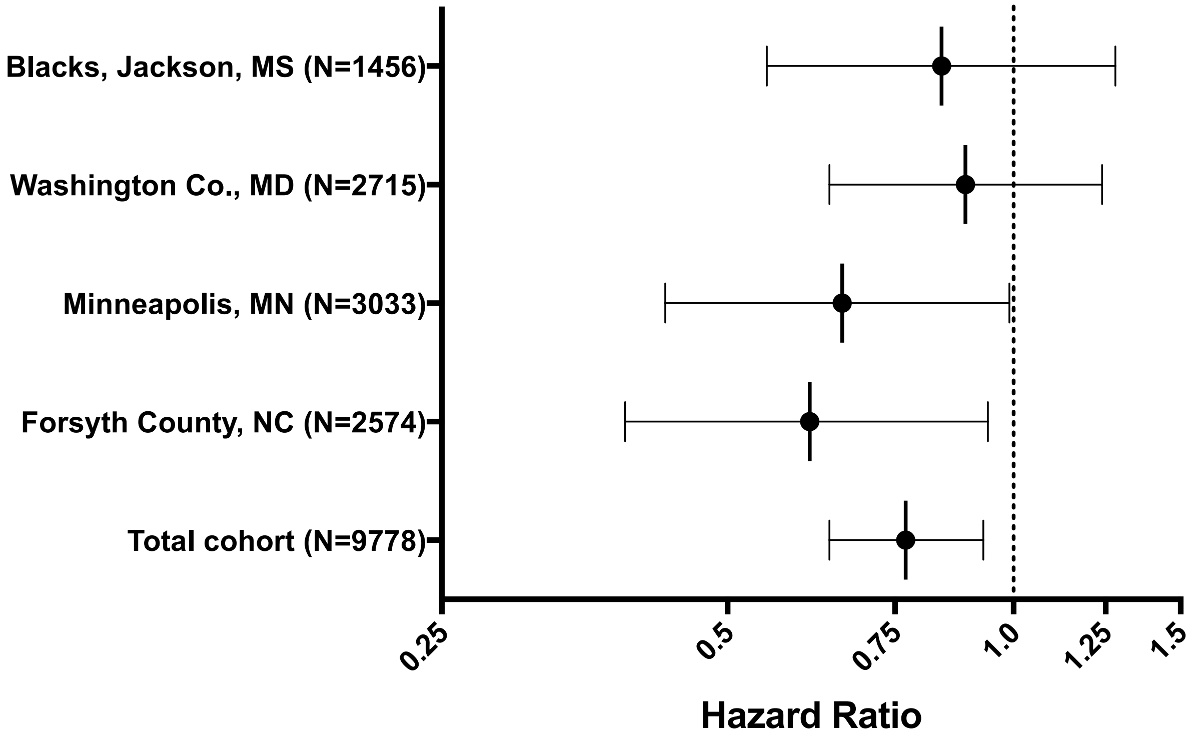

Adjusted for DM, BMI, Tchol, HDL, tobacco use and prior HF, genotype-adjusted log N-terminal B-type natriuretic peptide, and principle components; Stratified by age, sex, and race.
